# Transforming Food Systems: A Review of Sustainable Approaches to Minimize Food Loss and Waste

**DOI:** 10.1002/fsn3.71167

**Published:** 2025-11-11

**Authors:** Warren Kilemile, Kelvin E. Vulla, Fabian Mihafu, Vidhya Chandrasekaran

**Affiliations:** ^1^ Department of Food Science and Technology, College of Agricultural Sciences and Technology Mbeya University of Science and Technology Mbeya Tanzania; ^2^ Department of Food Science and Technology, College of Agricultural Sciences and Food Technology University of Dar es Salaam Dar es Salaam Tanzania; ^3^ Department of Food Packaging and Storage Technology National Institute of Food Technology, Entrepreneurship and Management, Thanjavur (NIFTEM‐T), Ministry of Food Processing Industries, Government of India Thanjavur Tamil Nadu India

**Keywords:** circular economy, digital platforms, food loss and waste, food security, food supply chain, food waste reduction, post‐harvest loss, smart logistics, sustainable food systems

## Abstract

Food loss and waste are significant global issues that have a profound impact on food security, the environment, and the economy. This review examines the magnitude, origins, and consequences of food loss and waste within the food system, emphasizing both technical and socio‐political dimensions. Evidence suggests that losses occur at multiple stages, including production, post‐harvest handling, retail, and consumption. The causes include inadequate infrastructure, poor handling practices, ineffective supply chains, consumer behaviors, and weak regulatory frameworks. High‐income nations generate more waste during consumption, whereas developing regions such as Sub‐Saharan Africa experience significant waste post‐harvest and throughout distribution. Existing interventions, including the expansion of the cold chain, utilization of digital platforms, and implementation of awareness campaigns, have proven to be inadequately scalable, particularly in resource‐constrained environments. Moreover, inadequacies persist in data quality, behavioral research, policy enforcement, and the incorporation of circular economy methodologies. This review is distinctive in that it examines the collective impacts on the environment, health, and economy, correlating them with governance and cultural influences, particularly in developing nations. The findings underscore the need for policies that are transparent, evidence‐based, and integrate food loss and waste reduction with national strategies for food security, climate change mitigation, and public health. To achieve SDG 12.3 and enhance the sustainability of food systems, it is crucial to strengthen data systems, promote private sector engagement, and foster innovations in the circular economy.

## Graphical abstract

This review highlights integrated strategies for minimizing food loss and waste across the global food supply chain. It identifies technological, behavioral, and policy‐driven interventions such as digital tracking, improved cold‐chain infrastructure, and circular economy innovations as critical to building sustainable food systems. Implementing these solutions can cut greenhouse gas emissions, conserve resources, enhance food security, and advance progress toward SDG 12.3.

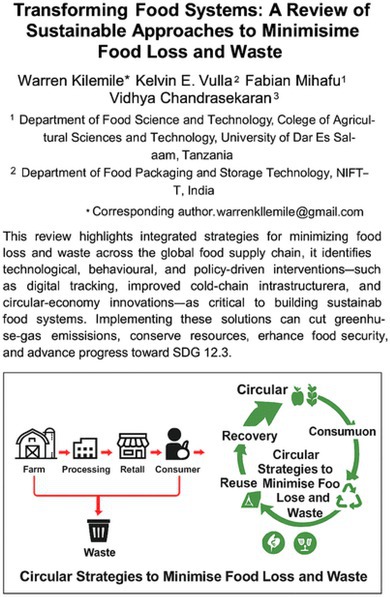

## 1 Introduction

Food systems (FS) worldwide face various challenges and have multiple objectives in response to those challenges. One such separate objective is to minimize food waste in the FS, which aligns with Sustainable Development Goal 12. It states that “ensures sustainable consumption and production patterns”. This goal includes a specific target (12.3) to halve per capita global food waste at the retail and consumer levels and reduce food losses along production and supply chains, including post‐harvest losses (UN, 2015) by 2030. The underlying reason for improving resilience in reducing food waste is to improve the environmental sustainability of food production (Bajželj et al. 2020). There is a common concern that food waste might threaten food security because of its vast amount and its impact on resource management (FAO 2017; Wang et al. 2021). The constant increase in the human population demands more food and products, piling up on agriculture and food waste. Waste generated from the food sector is primarily rich in proteins, carbohydrates, and lipids, which act as breeding grounds for numerous microorganisms (Sharma et al. 2021). A myriad of nutrients and high moisture content in food waste are the prime causes of dioxin formation during food waste incineration, leading to various health and environmental issues (Sharma et al. 2020).

Food waste can occur at every stage of the food supply chain, encompassing production, distribution, retail, and consumption. The absence of broadly accepted definitions of food waste (Lebersorger and Schneider 2011) complicates the study and quantification of food waste. Various classifications are produced on the basis of the contained resources, manufacturing methods, and management strategies (Elimelech et al. 2018). Table 1 provides a summary of previously employed definitions, highlighting various emphases. Multiple reports indicated that globally, one‐third of food produced for human consumption is lost, amounting to 1.3 billion tons annually, which was grown after utilizing 28% of the agricultural area, corresponding to the productivity of 1.4 billion hectares of fertile land all over the world (Sharma et al. 2020), and valued at almost US$1 trillion (FAO 2018; Mokrane et al. 2023). The annual cost of food waste, including social and environmental impacts, would approximate US$2.6 trillion. Approximately equal quantities of food are wasted in developed and developing nations, totaling 670 million tons and 630 million tons, respectively (Wong et al. 2022).

**TABLE 1:** Definitions of food waste.

Definition	Year	Emphasis	Author
Food waste is consumable food that remains uneaten, including items intended for human consumption but later lost, deteriorated, discarded, or contaminated	2021	Household consumer choices result in waste production	Attiq et al.
Food waste refers to food that is produced but not utilized, primarily because of domestic habits, dietary variety, and food preservation conditions in rural settings	2021	Waste from the viewpoint of a rural resident	Li et al.
Food waste refers to any food that is thrown away or lost at any point in the food system, resulting in excessive resource depletion and adverse environmental impacts	2021	The connection between food waste, nutrition, and sustainability	Conrad and Blackstone
Food waste refers to the portion of food intended for human use that is ultimately discarded instead of being consumed. It primarily occurs at the consumer and retail stages in developed nations	2018	Identifies the factors contributing to food waste to inform focused actions	Stangherlin and De Barcellos
Food waste, which includes consumable food discarded in hospitality establishments such as hotels, is primarily caused by inefficiencies in food management, unsustainable consumer practices, and inadequate trash disposal techniques	2019	Focuses on domestic practices and systemic shortcomings	Kasavan et al.
Food waste, either avoidable or unavoidable, occurs at various stages of consumption because of societal, personal, and cultural influences.	2018	Identifies the factors contributing to food waste to inform focused actions.	Stangherlin and De Barcellos
Food waste refers to food intended for human consumption that is ultimately discarded because of consumer behavior, including inadequate meal planning, misconceptions about food safety, and improper storage techniques	2018	Emphasizes the social, cultural, and material variables that affect garbage generation	Aktas et al.
Food waste is an unintended loss resulting from spoilage, inappropriate storage, and consumer interpretation of expiration dates	2017	Addresses both preventable and unavoidable waste	Hebrok and Boks
Food waste refers to food that is wasted, lost, or uneaten at any stage of the food distribution chain, encompassing manufacturing, processing, and final consumption	2016	Adheres to the European Union's waste management guidelines, encompassing various economic and environmental sectors	Canali et al.

The primary objective of this review was to investigate environmentally beneficial methods for reducing food loss and waste (FLW) in the global food chain. The review aims to consolidate existing knowledge on the causes, effects, and solutions to food waste, with a focus on approaches that have been proven effective. This effort aims to inform policymakers, researchers, and practitioners about the current state of FLW, the effectiveness of reduction measures, and the potential benefits of addressing it comprehensively. The review is also intended to serve as a starting point for future research and actions to achieve Sustainable Development Goal 12.3, which aims to halve global food waste by 2030. Moreover, the study aims to include specific instances and statistics from regional and national levels, focusing on both established and developing countries. The focus is on the whole food supply chain, from production and handling to retail and consumption. Urban markets, low‐income areas, and vulnerable groups are given special attention.

The main objectives of this review are as follows:

1. To examine how food waste affects the environment, the economy, society, and health.

The analysis examines how discarding food contributes to greenhouse gas emissions, depletes resources, incurs costs, and leads to food insecurity and starvation. It utilizes data from various regions, including Africa and Tanzania, to demonstrate how FLW impacts people in diverse social, economic, and ecological contexts.

2 To look at the benefit–cost ratio for minimizing global food waste and loss.

Reducing FLW seems to be economically profitable, environmentally essential, and socially justifiable. Although upfront costs are present, the return on investment is deemed overwhelmingly positive. The study will examine how FLW reduction is considered one of the most cost‐effective strategies for climate, development, and food security available today.

3 To discover how well different ways of cutting down on food waste work and their potential.

The evaluation examines various treatments, including new technologies, behavioral modification campaigns, legislative changes, and food redistribution systems. It also considers how likely they are to grow and have a lasting effect.

4 To highlight case studies, best practices, and lessons learnt worldwide.

We examine case studies from Europe, North America, Asia, and Africa to demonstrate the effectiveness of FLW programs and to generate valuable insights for implementing them elsewhere.

5 To find problems, challenges, and gaps in research and policy on food waste.

The evaluation highlights several key issues, including inadequate infrastructure, ineffective rule enforcement, insufficient consumer knowledge, and insufficient funding for innovative food system ideas.

## 2 Food Waste Versus Food Losses

Food waste and food losses are related but distinct concepts that pertain to the inefficient use of food resources. Food losses primarily occur earlier in the supply chain, often during production, post‐harvest handling, storage, and processing, and are typically due to factors such as pests, spoilage, poor infrastructure, or inadequate technology (Kaur and Watson 2024; Mesterházy et al. 2020; Stathers and Mvumi 2020). These losses result in edible food never reaching the consumer, thereby reducing the overall availability of food (Kaur and Watson 2024). In contrast, food waste typically occurs at the retail and consumer levels, often because of over‐purchasing, improper storage, expiration, or aesthetic standards that result in discarding edible food (Wright 2022; Urugo et al. 2024). Although both contribute to the unnecessary depletion of global food resources, losses are usually systemic issues related to supply chain inefficiencies, whereas waste often involves individual behaviors and choices.

## 3 Drivers of Food Waste

**FIGURE 1:**
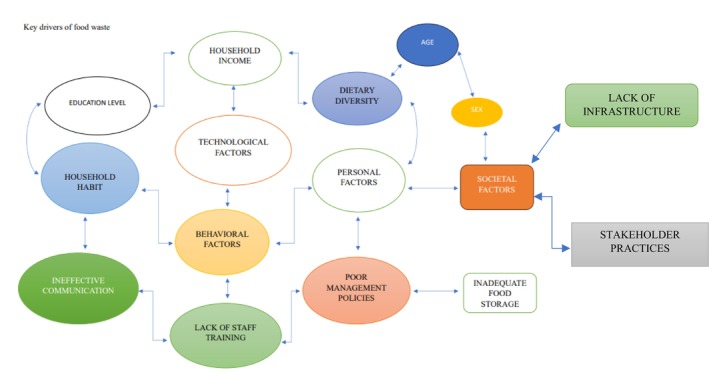
Key drivers of food waste.

Food waste emerges within various linked daily life practices, including shopping, storage, cooking, and consumption. Because of their entanglement in daily activities, consumers lack awareness of the multiple factors contributing to food waste. Sociological investigations of food waste explain the social organization of food practices within daily household activities and clarify how cultural, social, material, and temporal dimensions of food waste practices influence the perception of food as either edible or inedible (Figure 1), as well as the contextual approach to their study (Hebrok and Boks 2017). The material properties of food and the infrastructural conditions, including living circumstances, storage capacity, geographical proximity to retailers, and transportation options, significantly affect food waste by shaping daily routines (Soma 2020).

Decisions and actions regarding what and how much to purchase, the treatment of food before it reaches the consumer, its storage upon arrival at the household, and meal planning practices are key to reducing food waste (Liegeard and Manning 2020). The key drivers of food waste (Figure 1) can be explained differently on the basis of the emphasis on the definitions of food waste.

## 4 Education Level (Personal Factor)

Education is crucial in shaping people's awareness and attitudes toward food waste. Those with lower levels of education may not be fully informed about proper food storage, meal planning, or how to interpret food expiration labels, which often leads to unnecessary waste. A European survey found that over 60% of consumers discard food that is still safe because of misinterpretation of food labeling (Graham‐Rowe et al. 2014). In India, many rural households are unaware of refrigeration techniques, leading to fresh vegetables rotting quickly (Parfitt et al. 2010). A study by FAO (2019) showed that school programs incorporating food waste awareness in Brazil led to a 25% reduction in household food waste.

## 5 Household Habits (Behavioral Factor)

Daily household routines have a significant impact on food waste. Poor meal planning, over‐purchasing, and neglecting leftovers contribute significantly to the problem. A study conducted in the U.S. by Conrad and Blackstone (2021) proposed that supermarket promotions encourage bulk purchases, which often result in spoilage and increased food disposal. Also, a Canadian study found that one in four households regularly discards leftovers, significantly adding to food waste (Thyberg and Tonjes 2016). However, a mobile app in France that helps users plan meals and store food correctly led to a 30% reduction in food waste (Stenmarck et al. 2016).

## 6 Ineffective Communication (Societal Factor)

Gaps in communication, whether in supply chains, between retailers and consumers, or even within households, play a significant role in food waste. Gustavsson et al. (2011) reported that in Japan, a miscalculation in food orders for school cafeterias resulted in 20% of meals being discarded in 2018. Additionally, supermarkets in the UK discarded 3.6 million tons of edible food because of concerns about legal repercussions, rather than donating it. However, a well‐structured awareness campaign in Denmark led to a 25% decrease in household food waste in just 1 year (Stangherlin and De Barcellos 2018).

## 7 Poor Management Policies (Institutional Factor)

Inefficient retail, food services, and hospitality management policies contribute significantly to food waste. In Australia, nearly 30% of all harvested carrots are discarded simply because of minor imperfections (Parfitt et al. 2010). A study in Malaysia found that buffets generate up to 50% more waste compared to à la carte dining (Kasavan et al. 2022). In Germany, introducing tax incentives for food donations led to an 18% reduction in retail food waste (FAO, 2020).

## 8 Lack of Staff Training (Workforce Factor)

In many businesses and institutions, food waste occurs simply because employees are not adequately trained in handling food resources. In South Africa, 50% of post‐harvest fruit losses arise because of improper storage practices (Gustavsson et al. 2011). A hotel in Dubai introduced staff training on portion control, reducing food waste by 35% (Stenma), particularly in developing countries; this resulted in improved kitchen efficiency and cut food waste by 40% (Thyberg and Tonjes 2016).

## 9 Inadequate Food Storage Facilities (Technological Factors)

A lack of proper storage facilities, particularly in developing countries, results in significant food losses. In Nigeria, over 40% of tomatoes spoil before reaching markets because of inadequate cold storage (FAO 2019). In India, 20 million tons of wheat were lost in 2020 because of insufficient storage infrastructure (Parfitt et al. 2010). In Brazil, 30% of fish shipments are discarded because of transport delays (Graham‐Rowe et al. 2014).

## 10 Impacts of Food Waste

### 10.1 Environmental Impacts

Food waste is a significant contributor to environmental degradation worldwide, accounting for approximately 8%–10% of total human‐induced greenhouse gas (GHG) emissions (Table 2), primarily through methane emissions during decomposition in landfills (Hickey and Ozbay 2014). It is estimated that food waste in a nation would be the third‐largest GHG polluter after China and the United States (Lin et al. 2013; Shen et al. 2024). Food waste in the U.S. alone generates over 170 million metric tons of CO_2_ equivalent emissions yearly, up from 80 million tons, excluding gases from landfills (USDA 2022; Davis 2022). This environmental cost extends beyond GHG emissions, encompassing water wastage, land degradation, and biodiversity loss (Bhatt 2023). In sub‐Saharan Africa, 37% of the food produced per capita is lost annually, amounting to 120–170 kg per person, because of poor infrastructure, which also contributes to soil and water pollution (Mmereki et al. 2024; WRI 2023a). In Tanzania, nearly 3.5 million tons of food are wasted annually, contributing to an estimated 6 million metric tons of C0_2_ equivalent emissions, with 48% of food lost after harvesting never reaching the consumer. This waste significantly contributes to land and water contamination, and it also contributes to methane emissions, particularly in urban centers with poor waste management systems (MEDA 2023). Meanwhile, nations such as India waste 68 million tons of food annually, releasing 150 million tons of CO_2_ equivalent, whereas China leads with 91.6 million tons of food waste and emits 200 million tons of CO_2_ equivalent (FAO 2023, India; United Nations Development Programme 2023, China). Even middle‐income nations like Brazil waste 27 million tons, resulting in 55 million tons of CO_2_‐eq emissions (FAO 2023, Brazil). Nigeria wastes 38 million tons of food per year, resulting in 45 million tons of CO_2_‐Equivalent emissions, whereas South Africa discards 10.3 million tons, leading to 12 million tons of CO_2_‐equivalent emissions (WRI Africa 2023b; WWF South Africa 2017). Developed countries such as the United Kingdom and Germany waste approximately 9.5 million tons and 12 million tons of food, respectively, resulting in emissions of 25 million tons and 30 million tons of CO2‐eq (WRAP UK, 2023; European Commission 2023). Consequently, reducing food waste can directly mitigate environmental damage and promote sustainability.

**TABLE 2:** Status of food waste and GHG emissions in selected countries worldwide.

Country	Food waste (million tons/year)	Estimated GHG emissions (Mt CO_2_‐eq/year)	Source
United States	80	170	USDA (2022)
United Kingdom	9.5	25	WRAP UK (2023)
Germany	12	30	European Commission (2023)
India	68	150	FAO (2023) India
Nigeria	38	45	WRI (2023a) Africa
South Africa	10.3	12	WWF (2017) South Africa
Tanzania	3.5	6	MEDA (2023) Tanzania
China	91.6	200	United Nations Development Programme (2023) China
Brazil	27	55	FAO (2023) Brazil

### 10.2 Economic Impacts

Around the world, food waste generates economic losses of nearly $1 trillion annually (United Nations Development Programme 2023). In the United States alone, the annual value of food waste exceeds $218 billion, affecting farmers, retailers, and consumers (Ghosh et al. 2016). The European Union also incurs food waste–induced losses of up to €143 billion annually (European Commission 2023). Sub‐Saharan Africa loses over $4 billion annually because of post‐harvest losses resulting from poor storage, transportation, and market access (Stathers and Mvumi 2020). It directly affects food prices, farmer incomes, and economic growth. Therefore, reducing food waste presents an opportunity to enhance productivity and resilience in the agri‐food sector, thereby supporting economic development in low‐ and middle‐income countries.

### 10.3 Social Impacts

Food waste is a lost opportunity to end global hunger and promote food fairness. The world can produce food to feed 10 billion people, yet in 2023, nearly 735 million people were experiencing hunger (Hunger and Insecurity 2024). Conversely, almost one‐third of the world's food is lost or wasted (FAO 2023). The same imbalance is reflected in Africa, where 37% of food per person is wasted yearly, yet millions are food‐insecure (WRI 2023b). In Tanzania, particularly in the southern highland zone, food waste coexists with chronic undernutrition among children under five, despite high agricultural production. A study conducted reveals that substantial quantities of nutrient‐rich food are lost daily in the markets of Dar es Salaam, despite persistent food and nutrition insecurity. Nutrients lost through waste, including vitamin A and iron, could meet the daily needs of thousands, even as these nutrient deficiencies are common in Tanzania. This underscores a serious social issue—the loss of nutritious food that can help end hunger and malnutrition. Reducing food waste in retail could greatly enhance nutrition and public health. Thus, controlling food waste can contribute to social justice by bridging the gap between scarcity and plenty, promoting equal access to food.

### 10.4 Health Impacts

Food waste has indirect and direct health effects (Fami et al. 2019). Globally, the breakdown of organic waste in landfills releases methane, which contributes to climate change, exacerbating heat stress, respiratory diseases, and vector‐borne diseases (Aziz et al. 2020). In America, improper food waste disposal overwhelms municipal infrastructure and can lead to toxic leachates that contaminate groundwater beneath the land's surface. In Africa, the improper disposal of food waste contributes to environmental contamination, unsafe water supplies, poor sanitation, and increased exposure to diseases (International Water Association 2023). In Tanzania, over 70% of the diseases diagnosed at centers are water‐ and sanitation‐related, exacerbated by the decomposition of food waste and inadequate waste disposal systems (UNDP SDG Platform, 2023). This fact means that food waste reduction is not just a matter of sustainability and economics, but also a public health concern, particularly in urbanizing parts of Africa.

Therefore, food waste has a significant impact on the environment, economy, society, and public health. It drives greenhouse gas emissions, resource loss, and pollution, costing billions, especially in low‐income countries like Tanzania. Socially, it exacerbates hunger and malnutrition, despite the potential for wasted food to nourish thousands of people. The decomposition of waste also increases health risks. Addressing food waste through better infrastructure, education, and policy can benefit people, the planet, and economies.

## 11 The Role of Improper Stakeholder Practices and the Lack of Infrastructure

A primary contributor to FLW, particularly in developing countries, is inadequate infrastructure and the misconduct of stakeholders. Studies demonstrate that deficient infrastructure, characterized by insufficient cold storage, poor transportation systems, and limited access to sufficient market facilities, significantly intensifies post‐harvest and supply chain losses (Ali et al. 2021; Bappah and Adejoh 2024). Moreover, the infrastructural deficiencies are caused by insufficient or emerging governmental legal frameworks, characterized by enforcement and regulatory shortcomings that hinder the development of effective mitigation strategies.

Furthermore, alongside systemic barriers, inadequate practices by individuals within the supply chain also significantly contribute to food waste. Studies indicate that inadequate handling of produce, substandard packaging techniques, delayed harvesting, and insufficient knowledge or training among farmers can lead to food spoilage before it reaches consumers (Ali et al. 2021; Gouda and Duarte‐Sierra 2024; Osei‐Kwarteng et al. 2024). These practices reveal shortcomings at both the individual and institutional levels, as well as systemic flaws that sustain inefficiency.

In addition, the interplay between structural and behavioral factors indicates that a holistic approach is essential for addressing FLW (Surucu‐Balci and Tuna 2022). Reforming infrastructure challenges without improving stakeholder practices would result in negligible effects, similar to focusing exclusively on individual behavior without systemic improvements, thereby endangering sustainability. Therefore, to make a significant reduction in FLW within the food system, simultaneous enhancements in both infrastructure and stakeholder practices are essential.

## 12 The Global Benefit–Cost Ratio for Reducing FLW

A benefit–cost ratio is the ratio of financial benefits to financial costs attributable to the FLW actions or program (Clowes et al. 2019). Reducing global FLW presents substantial economic, environmental, and social benefits that far outweigh the costs of implementing reduction strategies (United Nations Environment Programme 2021; Luo et al. 2022). In other words, it can generate a triple win for the economy, for food security, and for the environment. From an economic perspective, the Food and Agriculture Organization (FAO) estimates that roughly one‐third of all food produced is lost or wasted, equating to around $1 trillion annually. This loss represents not only wasted food but also squandered labor, energy, water, and land resources (FAO 2023; Nénert et al. 2025). Investing in improved storage, transportation, processing, and market access, especially in developing countries, can significantly reduce post‐harvest losses and enhance food system efficiency (Goswami 2022).

Environmentally, reducing food waste is one of the most effective ways to lower greenhouse gas emissions. Decomposing food in landfills releases methane, a potent greenhouse gas, whereas the entire lifecycle of food from production to disposal contributes significantly to deforestation, biodiversity loss, and water use. Cutting FLW means less pressure to produce surplus food, thereby conserving ecosystems, reducing emissions, and mitigating climate change (Berry 2020; Hariram et al. 2023; FAO 2023). Furthermore, rechannelling surplus edible food toward food‐insecure populations enhances global food security without the need for expanding agricultural production. Socially, the benefits are similarly convincing. With hundreds of millions of people worldwide facing hunger or undernutrition, reducing food waste presents an opportunity to redistribute edible food to those in need. This can be facilitated through food banks, community kitchens, and food recovery networks. In this way, reducing food waste supports efforts to build more equitable, resilient, and sustainable food systems (FAO 2024; Sharma et al. 2024).

In July 2023, the European Commission (EC) proposed that Member States set legally binding targets to reduce food waste at the national level: 30% in retail, restaurants, food services, and households, and 10% in processing and manufacturing by 2030. The benefits of reducing food waste save the environment with a reduction of around 60 million tons of GHG emissions; savings of €400/year on average for a 4‐person household, which was significant in the 2023 context of high cost of living; and an overall value‐added to the EU economy of €1.6 billion (FAO 2023). Generally, across households, businesses, and farming systems, the economic and environmental returns enormously outperform the costs. Beyond financial benefits, FLW reduction helps combat climate change, supports food security, and fosters equitable, resilient food systems.

The global benefits and costs of reducing FLW vary significantly across regions, with notable implications for greenhouse gas emissions, food availability, and economic efficiency. As shown in Table 3, East Asia and the Pacific have the highest potential for emissions reduction (250 Mt. CO_2_‐eq/year). At the same time, Sub‐Saharan Africa stands out for its food security potential, with the capacity to feed an additional 120 million people annually.

**TABLE 3:** Regional benefits of reducing food loss and waste.

Region	GHG reduction (Mt CO_2_/year)	Food saved (Mt/year)	Economic savings (billion USD/year)	People fed (millions)	Source
North America	180	60	200	60	WRI (2023b)
Europe	200	70	220	70	FAO (2023)
Sub‐Saharan Africa	80	45	80	120	United Nations Environment Programme (2021)
South Asia	90	50	90	140	FAO (2023)
East Asia and the Pacific	250	95	300	100	WRI (2023b)
Latin America and the Caribbean	150	55	150	90	United Nations Environment Programme (2021)
Middle East and North Africa	70	30	70	60	FAO (2023)
Global	1000	405	1110	640	Combined Global Estimates (2023)

One of the most profound benefits of reducing FLW is its potential to mitigate climate change. Food systems contribute up to 30% of global greenhouse gas (GHG) emissions (Crippa et al. 2021); FLW alone accounts for 8%–10% of these emissions annually (Higuera‐Ciapara et al. 2020). By preventing food from being produced and wasted, emissions associated with land use change, fertilizer application, transportation, refrigeration, and waste decomposition are significantly reduced (Bhatia et al. 2023). This has led to widespread agreement that FLW reduction is among the most cost‐effective climate mitigation strategies, especially compared to more capital‐intensive technologies (Galford et al. 2020).

In parallel, reducing food waste contributes to the conservation of natural resources, including land, water, and biodiversity. Food production is a leading driver of global deforestation and freshwater use (Wunderlich and Martinez 2018). For instance, it is estimated that nearly 30% of agricultural land is used to grow food that is ultimately never consumed (FAO 2023). Reducing FLW can help alleviate the demand for new cropland and mitigate pressure on critical ecosystems, such as rainforests, wetlands, and fisheries. It also contributes to water security by alleviating the water‐intensive nature of food systems, particularly in water‐stressed regions (Wunderlich and Martinez 2018).

Studies have shown that businesses often experience a high return on investment when implementing waste‐reduction strategies, with an average benefit–cost ratio of 14:1 in some sectors. Thus, for every $1 (or other relevant currency) invested in FLW reduction, half of the surveyed company sites realized a return of $13 or greater (Clowes et al. 2019; Atz et al. 2020; Liu and Li 2023).

Therefore, the global benefits of reducing FLW are extensive and interconnected. From lowering emissions and conserving resources to promoting food security and enhancing economic efficiency, FLW reduction is vital to achieving a more sustainable and resilient global food system. Unlocking these benefits requires coordinated actions across sectors and scales supported by robust data, inclusive governance, and investment in innovation and education.

## 13 Impact of FLW Reduction on the Four Dimensions of Food Security and Global Nutrition

Enhancing global nutrition and food security is crucial for promoting sustainable development and improving the well‐being of people worldwide (Pérez‐Escamilla 2017). As the global population continues to grow, and in light of climate change and increasing urbanization, ensuring that every individual has consistent access to sufficient, safe, and nutritious food is more crucial than ever. Innovations in agricultural practices, such as climate‐smart farming and the adoption of resilient crop varieties, can significantly increase food production in vulnerable regions (Makate et al. 2019). Moreover, reducing food waste, promoting equitable distribution, and implementing social safety nets are essential steps to guarantee that marginalized communities have reliable access to adequate and nutritious food. Educational campaigns focusing on healthy eating habits and nutritional awareness can further empower individuals to make informed dietary choices. By enhancing global cooperation, investing in research and infrastructure, and prioritizing sustainable practices, we can strive for a future in which no one suffers from hunger or malnutrition, ultimately contributing to healthier and more resilient societies worldwide.

Food security is defined in terms of four key dimensions: availability, access, utilization, and stability. Reducing FLW has a significant and multidimensional impact on global food security. Addressing FLW contributes positively to each of the dimensions, making it a strategic priority for improving food systems worldwide. Generally, FLW contributes to the reduction of food access and availability, hence worsening food security (Corrado et al. 2019).

Food availability can be improved by reducing FLW, mostly by increasing the overall supply of edible food without requiring additional agricultural production. Of this, about one‐third of all food produced globally (approximately 1.3 billion tons) is lost or wasted each year. Therefore, recovering even a portion of this food can significantly boost supply, mainly in regions where food is lost during production, storage, and distribution because of inadequate infrastructure/technology (FAO 2017; Santeramo and Lamonaca 2021).

FLW reduction improves food access, particularly for low‐income populations. Increasing food supply and improving efficiency across the supply chain help to lower prices and stabilize markets. On the other hand, reducing FLW contributes to better food utilization by preserving food nutritional quality and safety. Additionally, improving food handling, storage, and consumer education reduces spoilage, ensuring that nutritious food reaches people in good condition. This supports better health outcomes through increased intake of safe foods and balanced diets (Corrado et al. 2019; Santeramo and Lamonaca 2021).

The stability of food systems can be strengthened by reducing FLW, by making them more resilient to shocks such as price fluctuation, supply chain disruptions, and climate change. This enhances long‐term food security and sustainability (FAO 2017). In conclusion, minimizing FLW is an effective strategy for improving food security in all its dimensions, ensuring that more people have consistent, affordable access to safe and nutritious food, both now and in the future.

## 14 The FLW Accounting and Reporting Standard (FLW Standard)

According to Hanson et al. (2016), the FLW accounting and reporting standard (FLW standard) is a global framework that provides requirements and guidelines for measuring and reporting the weight of food and associated inedible parts removed from the food supply chain. This is commonly referred to as “food loss and waste”. By utilizing this standard, countries, cities, companies, and other entities can create inventories to track the amount of FLW generated and its ultimate destination. These inventories can help to inform and guide strategies aimed at reducing FLW. Minimizing FLW can lead to economic benefits, enhance food security, improve the efficiency of natural resource use, and reduce environmental impacts (FAO 2019).

The key strengths and contributions of the FLW standards include standardization and comparability, which provide a unified approach that facilitates comparisons across various regions and sectors (Hanson et al. 2016). This consistency supports benchmarking and allows for the tracking of progress over time, both of which are essential for effective policy formulation and industry accountability. Another significant advantage is the comprehensive scope of the FLW standards, which covers all stages of the supply chain from production and handling to processing, distribution, retail, and consumption, ensuring a holistic understanding of FLW (Luo et al. 2021; Heydari 2024). Additionally, the flexibility and scalability of the FLW standards enable them to be used by a wide range of entities, from small farms to large corporations and government bodies. This versatility allows for diverse applications tailored to the available data and resources (Nicastro and Carillo 2021). Ultimately, the standards foster data‐driven decision‐making by offering clear metrics and methodologies, which encourage evidence‐based strategies for minimizing FLW. This can lead to more targeted and effective interventions (Omolayo et al. 2021; Akkerman and Cruijssen 2024).

Despite its comprehensive framework, the practical application of measuring FLW can be obstructed by challenges such as data availability, quality, and resource limitations, particularly in developing countries or smaller operations (Gage et al. 2024; Ishangulyyev et al. 2019). Accurately measuring FLW necessitates considerable effort and capacity‐building. Furthermore, the intricate methodology may be overwhelming for organizations lacking the necessary technical expertise or robust data infrastructure, potentially resulting in inconsistent application or superficial compliance. Another concern is the potential for under‐reporting; as with many self‐reporting standards, there exists a risk of misreporting because of insufficient verification mechanisms or a lack of incentives to report improved performance. Additionally, although the standard offers useful measurement tools, it may not adequately address the cultural, behavioral, and socioeconomic drivers of food waste, which are essential for designing effective interventions. Finally, although quantification is critical, relying solely on measurement does not ensure reduction; there is a pressing need to complement the standard with actionable policies and strategies focused on encouraging behavioral change.

Therefore, the FLW standard represents a significant advancement in establishing a unified language and methodology for addressing FLW on a global scale. Its focus on standardization and its comprehensive scope render it an invaluable resource for stakeholders committed to achieving sustainability objectives. However, its effectiveness depends on widespread adoption, capacity‐building, and alignment with broader strategies that address the root causes of FLW. To enhance its impact, stakeholders should consider pairing measurement efforts with targeted interventions, policy support, and initiatives to overcome operational and cultural hurdles.

## 15 Food Waste Reduction and Sustainable Food Systems

In the face of mounting environmental challenges, social inequalities, and economic pressures, the concepts of reducing food waste and promoting sustainable food systems have garnered increasing global attention. Addressing food waste is not only vital for conserving resources but also integral to establishing resilient and equitable food systems. A sustainable food system delivers food security and nutrition for all while ensuring environmental health, economic viability, and social equity (Capone et al. 2014; El Bilali et al. 2019). It encompasses the entire food value chain, from production and processing to distribution, consumption, and waste management, guided by principles that promote ecological balance, efficient resource use, and social justice (Van Berkum et al. 2018). The critical components of sustainable food systems are environmental sustainability, economic viability, social equity, resilience, and adaptability.

- Ecological sustainability

Ecological sustainability emphasizes the importance of minimizing environmental impacts associated with food production, such as pollution, habitat destruction, and contributions to climate change (Munang et al. 2011). Implementing environmentally friendly agricultural practices such as crop rotation, organic farming, and reduced pesticide use can significantly improve soil health and promote biodiversity (Gomiero et al. 2011; Tahat et al. 2020). However, the widespread adoption of these practices faces challenges, particularly in terms of economic limitations and knowledge gaps among farmers, especially in developing regions. Overcoming these barriers is essential for fostering a sustainable agricultural landscape.

b Economic viability

Economic viability focuses on ensuring that the processes of food production and distribution are both profitable and accessible to all stakeholders (Oliver et al. 2018). Although economic incentives can drive innovation and enhance operational efficiency, they can also lead to issues such as overproduction or reliance on monoculture practices, which may disrupt ecological balance. Achieving a delicate equilibrium between profitability and sustainability poses a critical challenge that must be addressed to develop resilient food systems (Agarwala et al. 2022; Oliver et al. 2018).

c Social equity

Social equity seeks to ensure fair access to nutritious food while fostering equitable labor practices throughout the food supply chain (Onyeaka et al. 2024). Tackling challenges such as food desert areas with limited access to affordable and healthy food and guaranteeing fair wages for workers is crucial in creating a more equitable food system. Nevertheless, systemic inequalities often persist, hindering progress toward social sustainability.

d Resilience and adaptability

Resilience and adaptability refer to the ability of food systems to withstand shocks, such as climate change, pandemics, or market fluctuations (Kahiluoto 2020; Béné 2020). Strategies like crop diversification and localizing food production can enhance the resilience of these systems, making them better equipped to respond to unforeseen challenges (Béné 2020). However, these approaches may conflict with existing global trade models, complicating their implementation. It is essential to strike a balance between local resilience and global economic integration to create robust food systems that can adapt to changing circumstances.

Reducing food waste stands as a pivotal goal in the quest to strengthen sustainable food systems. It involves implementing various strategies that tackle the multiple nature of food waste from production to consumption.

The primary strategy focuses on improving supply chain management, which involves enhancing forecasting, storage, and transportation processes to reduce spoilage. Effective supply chain management is particularly important in developing countries, where inadequate infrastructure often increases food loss (Khan et al. 2022; Heydari 2024). Investments in advanced cold storage facilities and efficient logistics can significantly reduce waste. However, there are significant challenges to overcome. High infrastructure costs and limited access to technology can create substantial barriers, making it difficult for these regions to implement effective systems (Arowosegbe et al. 2024). Therefore, although improving supply chain management is essential, it requires coordinated efforts and resources to address these challenges.

Another strategy involves consumer education and behavior change. Raising awareness about the effects of food waste and promoting mindful consumption practices are essential for shifting societal attitudes toward food (Morkunas et al. 2024; Mohammad and Quoquab 2024). Educational campaigns that inform consumers about food waste statistics and sustainable practices can play a pivotal role in altering behaviors (Redman and Redman 2014). However, the success of these campaigns relies on cultural sensitivity and targeted messaging. Different communities may possess unique perspectives and habits related to food consumption and waste. Therefore, understanding these cultural nuances and designing tailored educational programs is crucial for fostering genuine change in behavior regarding food waste.

The implementation of innovative preservation technologies plays a crucial role in minimizing food waste. Techniques such as vacuum packaging, fermentation, and dehydration can greatly extend the shelf life of various food products, thereby reducing spoilage (Sridhar et al. 2021). Although these technologies present significant potential, they often demand substantial upfront investments and a readiness from consumers to embrace new methods of food preservation. This poses a dual challenge: businesses must not only invest in these technologies, but there also needs to be consumer education aimed at fostering acceptance and understanding of their benefits.

Moreover, implementing strategies for food redistribution and donation is essential in tackling the issue of food waste. Redirecting surplus food to food banks or charitable organizations not only helps alleviate hunger but also reduces waste (Karki et al. 2021; Galli et al. 2019). However, this approach often faces legal and logistical challenges that can hinder its effectiveness. It is crucial to ensure the safety and dignity of recipients, and navigating the regulations related to food safety can be quite complex (Karki et al. 2021). Consequently, developing streamlined processes and policies that facilitate food donation while ensuring safety is a significant challenge that must be addressed.

On a broader scale, policy and legislation are crucial in the collective effort to mitigate food waste. Implementing regulations, such as reforming date labeling practices and introducing waste taxes, can effectively incentivize waste reduction across various levels (Chenavaz and Dimitrov 2024). However, the success of these policies relies heavily on their enforcement and the collaboration of diverse stakeholders. Policymakers must remain vigilant regarding potential unintended consequences, such as the risk of transferring food waste to other sectors, which could ultimately undermine the overarching objectives of waste reduction initiatives.

## 16 The Role of Public Campaigning in Reducing FLW Among Students and in Hotels

Public awareness campaigns have emerged as essential strategies to reduce FLW, particularly within educational institutions and the hospitality sector. These campaigns aim to influence consumer behavior by educating individuals, engaging them, and promoting the adoption of environmentally sustainable practices. Targeted awareness campaigns have significantly reduced food waste in university environments. Studies conducted in universities show that the implementation of informational signs, such as stickers providing food ordering tips and cards detailing resource utilization, nearly doubled the proportion of students who completed their meals (da Costa et al. 2022; Fraj‐Andrés et al. 2023; Kirshnan et al. 2024). This intervention significantly reduced the types of food waste generated. Additionally, a study by Yu et al. (2023) demonstrates that the “FoodWise” initiative at The Hong Kong University of Science and Technology employed a dual‐system approach that integrated data visualization and gamification. Furthermore, over 200 individuals participated in a 2‐week duration, recording in excess of 800 daily actions aimed at food conservation. This indicates that individuals are increasingly aware of and modifying their behaviors to minimize food waste.

In addition, public campaigns have proven effective in the hospitality industry. A study by Dolnicar et al. (2020) found that the implementation of communication tools on hotel guest tables resulted in a 34% reduction in plate waste. Also, organizing information effectively can help guests make more informed dietary choices. Campaigns, both domestically and internationally, have facilitated greater awareness of FLW. The “Love Food Hate Waste” campaign, launched by the Waste and Resources Action Programme, has played a pivotal role in informing the public about strategies for reducing food waste (Jin and Nichols 2020; Saner 2023). The campaign succeeded because of widespread participation and its significant impact on behavior.

Therefore, a critical interconnection exists between the reduction of food waste and the sustainability of food systems. By conserving resources, diminishing greenhouse gas emissions, and addressing food insecurity, waste reduction fortifies the overall resilience of food systems. Sustainable food systems inherently strive to minimize waste through practices that embody environmental, public campaign, economic, and social considerations. Economic incentives frequently prioritize overproduction, culminating in surplus and waste, particularly at the agricultural level. Furthermore, entrenched consumer behaviors can be resistant to transformation, and developing regions may grapple with infrastructural inadequacies that hinder the enhancement of cold chains. Consequently, a comprehensive strategy that amalgamates technological innovations, adaptive policy frameworks, educational initiatives, and cultural shifts is imperative for effectively combating food waste and nurturing sustainable food systems.

## 17 Food Waste Reduction Potential

The increasing need to tackle food waste globally, regionally, and nationally has given rise to various strategies for minimizing losses across the food supply chain. The interventions for reducing food waste work depend upon the environment in which the intervention is made, the intensity of stakeholder involvement, baseline infrastructure, application of policies, and societal attitude toward the value of food and the culture of consumption. This section discusses the potential of the various domains of intervention in contributing toward the agenda of sustainable development of the SDGs, especially SDG 12.3 of halving per capita global food waste at the retail and consumer levels by 2030 (FAO 2023).

To begin with, consumer awareness and behavioral change campaigns are the most successful strategies to address household food waste. Such programs are based firmly on disseminating information and public involvement, aiming to enhance food management using meal planning, proper food storage, and decoding date labeling (Kim et al. 2020; Soma et al. 2020; Vittuari et al. 2023). Studies have demonstrated that the awareness of the economic and environmental implications of food waste among consumers leads them to change their behavior toward better consumption (Stancu et al. 2016). Education and social marketing support this behavioral change and result in a substantial reduction of food waste in households in high‐income environments (United Nations Environment Programme 2021).

In addition, food recovery and redistribution are other key areas of influence, which entail directing excess food away from manufacturers, retailers, and distributors to institutions that feed food‐insecure individuals. These initiatives have had dual advantages: they have reduced avoidable waste and improved access to food. A study by Patel et al. (2021) concluded that food waste reduction and food poverty alleviation can work together by recovering and redistributing excess food, which can effectively eliminate the production of preventable food waste and solve food poverty simultaneously. Moreover, redistribution schemes are wildly successful in cities with excess and demand in neighboring regions (Karki et al. 2021). They rely on effective logistics, favorable legislative environments, and collaboration among food businesses, charities, and cities (FAO 2019).

Furthermore, technological advancements drive the minimization of food waste in all sectors. Utilizing digital technologies to track inventory, forecast spoilage, and distribute excess food maximizes operational efficiency and waste minimization. Artificial intelligence (AI), the Internet of Things, and applications enable stakeholders to trace food flows in real time, enhancing decision‐making and minimizing overproduction and loss (Anwar et al. 2023; Elgalb and Gerges 2024). Despite this, access to these technologies is uneven, particularly in low‐income countries, making them less scalable and efficient in these regions.

Another key aspect is that post‐harvest handling and storage are key to minimizing losses in low and middle‐income countries. Investment in cold chain infrastructure, better packaging, and efficient transport mechanisms minimizes spoilage risk before reaching consumers (Pajic et al. 2024). In many cases, these measures' success depends on access to electricity, roads, and institutions. In hot climates and poorly developed logistics systems, even modest gains in post‐harvest management can lead to significant food loss reductions (FAO 2023).

On a different note, measuring and tracking food waste through the complete value chain of the food system is of equal significance (Economou et al. 2024). This strategy enables stakeholders to gauge the quantity and channels of waste, establish achievable goals, and monitor developments. Although this strategy is tremendously potent in informing policy and investment, it demands institutional capacity, standardization of the measurements, and implementation of national or regional systems of food waste accounts (United Nations Environment Programme 2021).

Equally important, the circular economy system presents a complementary approach to reducing food waste by encouraging waste recycling into animal feed, compost, and bioenergy. These methods reduce environmental pollution and extract economic value from waste flows. Although the systems are cost‐efficient and have sustainability objectives, they may need the inputs of technologies and the regulatory support of governments to secure safety and quality assurance when recycling back into the chain of feed or food.

Moreover, public policies and regulatory instruments play a pivotal role in determining the success of food waste interventions (Aramyan et al. 2021; Cattaneo et al. 2021). Policies incentivizing food donation, penalizing excessive waste, or mandating waste reporting can drive systemic change (Ryen and Babbitt 2022). However, the effectiveness of such regulations depends on enforcement mechanisms, clarity of mandates, and the provision of support systems for compliance. In countries with strong governance and food safety systems, legal instruments have been a key driver of reduced food waste (European Commission 2023).

Finally, the hospitality and food service industry holds considerable potential for food waste reduction. Amicarelli et al. (2022) proposed that there are many chances for the hotel industry to cut down on food waste. However, both individual establishments and government agencies must make several efforts. The current study demonstrates that managers, chefs, and service personnel in Romania and Italy are cognizant of the problems associated with food waste from a financial, social, and environmental standpoint and are constantly seeking solutions. However, they face daily challenges in reducing it (Amicarelli et al. 2022). Also, interventions in this sector typically involve staff training, portion size adjustments, buffet redesign, and back‐of‐house audits (Tuan et al. 2024). These measures are effective because they directly address where food is most commonly wasted because of over‐preparation or lack of demand forecasting. Their success relies on managerial commitment, staff awareness, and customer participation in reducing plate waste (HLPE, 2020).

The effectiveness of food waste reduction strategies is multifactorial and context‐specific. Although individual approaches can yield measurable benefits, their full potential is realized when combined with supportive infrastructure, robust policy frameworks, public engagement, and technological innovation. Holistic and cross‐sectoral strategies remain the most effective pathway to meaningful and sustained reductions in food waste.

## 18 Enabling a Circular Economy to Fight Food Waste

One of the biggest causes of waste production in the world is the food business. Food waste, packaging trash, and processing waste are among the many waste products produced during the production, processing, and distribution of food products. Waste management in the food sector has grown to be a crucial problem since it has an impact on the environment as well as the economy and society (de Almeida Oroski and da Silva 2023). Waste management presents a number of difficulties for the food business. The first difficulty is the enormous amount of garbage produced. FAO highlights that approximately one‐third of the world's food production for human consumption (roughly 1.3 billion tons annually) is lost or wasted (WWF‐UK 2021). This waste contributes to water pollution, soil degradation, and greenhouse gas emissions, all of which have a major effect on the environment. The cost of waste handling is the second issue (Stancu et al. 2016).

Transportation, processing, and landfill or incinerator facilities are among the major resources needed for garbage disposal. Customers frequently pay higher prices for food products as a result of these costs being passed on to them. The societal impact of waste is the third difficulty. Because it decreases the amount of food available to those in need, food waste has a major effect on food security. Furthermore, the livelihoods of people who rely on natural resources like land and water are impacted by food waste's contribution to their depletion (Garrone et al. 2014). The food business must implement circular economy techniques for sustainable waste management in order to overcome these obstacles. According to the circular economy concept, materials should be reused, repurposed, and recycled in order to prolong their useful life (Beretta et al. 2013).

The circular economy concept emphasizes waste reduction and resource efficiency, which can have major positive effects on the economy, society, and environment. There are several ways to implement circular economy techniques for sustainable waste management in the food sector (Spang et al. 2019). Improving food preservation methods and putting in place more effective manufacturing and distribution systems are two ways to cut food waste at its source. Reusing waste resources, such as turning food scraps into animal feed or composting them to make fertilizer, is an additional strategy. Creating more recyclable and sustainable packaging is another circular economy tactic (Schmidt and Matthies 2018).

Using biodegradable materials, cutting back on packing, and creating packaging that is simpler to recycle are a few examples of this. Lastly, creating new business models that promote more environmentally friendly practices can also be a part of circular economy initiatives. Some businesses are experimenting with circular supply chains, for instance, in which goods are made to be readily disassembled and reused at the end of their lifecycle (Reynolds et al. 2019). Other businesses are looking at new ways to generate income by converting garbage into goods with added value, such as using food waste to make bioplastics or biofuels.

In order to guarantee sustainability over the long term, the food industry must tackle the problem of waste management. With its emphasis on resource efficiency, waste reduction, and the creation of new economic opportunities, the circular economy offers a framework for sustainable waste management. The food business may lessen its impact on the environment, cut expenses, and help create a more sustainable future by implementing circular economy practices (Stöckli et al. 2018).

## 19 Circular Economy Concept

In response to the pressing need for more environmentally friendly waste management techniques, the idea of the “circular economy” has drawn increased attention in recent years. An economic concept known as the “circular economy” seeks to reduce waste and maximize the use of resources by extending their useful lives. Reducing, reusing, and recycling resources and materials to build a closed‐loop system that minimizes waste and keeps commodities in use for as long as feasible are its guiding principles (De Bernardi et al. 2023).

This approach is intended to lessen the adverse effects of human activity on the environment and encourage the wise use of natural resources. Circular economy techniques can help the food business in many ways, from lowering greenhouse gas emissions and food waste to generating new income and enhancing brand recognition. An outline of the circular economy concept, its tenets, and the benefits of implementing it in food sector waste management procedures will be given in this article (Puram and Gurumurthy 2023).

## 20 The Circular Economy: Definition and Foundations

A circular economy is a system of economics that is regenerative and restorative by nature. This economic model seeks to minimize pollution and waste while extending the useful life of materials. The ‘take‐make‐dispose’ method of the conventional linear economy is an alternative to this one. “An economy that is restorative and regenerative by design, aiming to keep products, components, and materials at their highest utility and value at all times, distinguishing between technical and biological cycles,” is how the Ellen MacArthur Foundation, a principal proponent of the circular economy, defines it (Cong et al. 2019). The circular economy encourages the effective use of natural resources, lowers waste production, and prolongs the life of materials and products in an effort to close the loop of the conventional linear economy. By minimizing waste and conserving, reusing, and recycling resources, it seeks to establish a closed‐loop system (Tromp et al. 2016).

The concept is based on three main principles:

1. Preventing pollution and waste;
2. Reusing resources and goods; and
3. Restoring natural systems.

The primary goal of the first principle is to stop waste and pollution before they start by creating more durable, recyclable, and efficient goods and procedures. The significance of creating sustainable and eco‐friendly systems and goods is emphasized by this principle. This entails adopting a more circular model of production and consumption, where resources are recycled and reused, as opposed to the conventional linear model, which extracts, uses, and discards resources (Luu 2021). It entails applying eco‐design, which emphasizes how systems and products affect the environment from the time of conception until the end of their useful lives. We can lessen the adverse effects of human activity on the environment by planning out waste and pollution. The second principle encourages reuse, repair, and recycling in order to highlight the significance of extending the useful life of materials (Aschemann‐Witzel et al. 2017). The “reduce‐reuse‐recycle” strategy, which minimizes waste and keeps resources in use, replaces the “take‐make‐dispose” strategy. We can lessen waste production and preserve natural resources by reusing products and materials (Kannan et al. 2024). The third principle emphasizes the necessity of regenerating natural systems through the design of restorative and regenerative processes as opposed to polluting and degrading ones. In order to minimize waste and restore natural systems, this entails moving away from a linear model of production and consumption and toward a closed‐loop system (Aschemann‐Witzel 2018). It entails the application of sustainable methods, including sustainable forestry and agriculture, to guarantee the resilience and well‐being of ecosystems (Attiq et al. 2021).

## 21 Benefits of the Circular Economy Approach to Food Industry Waste Management

There are several benefits to using circular economy techniques in food sector waste management procedures, such as:

1. Minimizing food waste

A third of the world's food production is thought to be lost or wasted annually, making the food business a significant contributor to food loss worldwide. In addition to removing food waste from landfills and turning it into new goods like compost or animal feed, circular economy initiatives can also assist in minimizing food waste by encouraging more efficient production and distribution networks (Lapidge 2020). This encourages the effective use of natural resources and prolongs the life of materials and products, which helps to reduce waste. We can lessen the adverse effects of human activity on the environment and preserve natural resources by cutting back on waste (Adenso‐Díaz et al. 2017).

2 Reduced emissions of greenhouse gases

Food waste contributes to climate change by being a major source of greenhouse gas emissions. As food waste breaks down in landfills, methane, a powerful greenhouse gas that traps heat in the atmosphere around 25 times more effectively than carbon dioxide, is released. Food waste accounts for about 8% of greenhouse gas emissions worldwide, according to the UN (Bolwig et al. 2021). Greenhouse gas emissions in the food business can be considerably decreased by putting circular economy principles into practice, such as minimizing food waste, recovering food for human use, and turning food waste into energy (Berger 2014). Emissions can also be decreased by encouraging the use of renewable energy sources, such as wind or solar, in the processing, transportation, and production of food. In general, reducing the consequences of climate change can be greatly aided by actions taken to eliminate food waste and encourage sustainable practices in the food business (Calic and Mosakowski 2016).

3 Developing new sources of income

Through the identification of value in waste materials and by‐products, the implementation of circular economy methods can assist companies in the food industry in discovering new avenues for revenue production. Businesses can produce new energy sources and sell them to consumers by turning food waste into biogas or biofuels, which will increase their revenue streams. Businesses can use waste materials to produce new goods and services in addition to energy (Dzhengiz et al. 2023). Food waste, for instance, can be turned into animal feed or fertilizer that farmers can purchase. As an alternative, food waste can be transformed into new culinary items that can be sold to customers, like jams or sauces. Businesses can use these products as an extra selling factor by marketing them as eco‐friendly and sustainable. Businesses in the food sector can increase sustainability and reduce waste by adopting circular economy tactics. They can also open up new revenue streams and growth prospects (Belk 2007).

4 Enhancing brand image

Businesses in the food sector can improve their brand reputation among stakeholders and consumers by showcasing their dedication to sustainability and environmental responsibility through the use of circular economy methods. Since more and more customers are growing environmentally conscious and actively looking for goods and services from businesses that prioritize sustainability, this is especially crucial. Employing circular economy techniques like employing renewable energy sources, cutting down on food waste, and adopting sustainable procurement methods can help companies show their dedication to sustainability and lessen their environmental impact (Apostolidis et al. 2021).

This may consequently result in a boost in client loyalty, a better reputation for the brand, and an improvement in the bottom line. In the current business climate, where businesses are expected to operate in an environmentally responsible manner more and more, adopting circular economy strategies can help businesses in the food industry not only improve sustainability and reduce waste but also improve their reputation and attract customers who value sustainability (Weymes and Davies 2019).

5 Preservation of natural resources

In the food business, circular economy practices can aid in the preservation of natural resources by encouraging resource efficiency and cutting waste. In order to lessen the environmental impact of food production, for instance, sustainable sourcing methods can minimize the use of natural resources like energy and water. Additionally, minimizing food waste can contribute to resource conservation by preventing the waste of resources used in food production. In addition to encouraging material reuse and recycling, circular economy techniques can help preserve natural resources (Morone et al. 2019).

For instance, food scraps can be turned into compost, which can be applied to crops as a natural fertilizer. Natural resources can be preserved, and fewer new materials are needed when food packaging materials are recycled or repurposed. Businesses may ensure the availability of resources for future generations by implementing circular economy ideas in the food industry, which will also help to conserve natural resources, minimize waste, and promote sustainability (Choi et al. 2019).

6 Employment creation

The food business can provide new employment possibilities by implementing circular economy techniques, especially in the recycling, repair, and refurbishing sectors. For instance, turning food waste into biogas or biofuels necessitates specific tools and knowledge, opening up new career prospects in this field. In a similar vein, new jobs may be generated by the maintenance and repair of machinery used in food processing and packaging. Strategies for the circular economy can also lead to the creation of new jobs in the fields of sustainable production and sourcing (Guyader 2018). Companies that place a high priority on sustainable sourcing methods, for instance, would need to employ specialists in sustainable forestry or agriculture. In a similar vein, companies that use renewable energy sources could need to employ personnel with specific knowledge in this field. Businesses can generate new job possibilities by adopting circular economy tactics in the food industry, especially in local regions where these jobs are most needed. This might boost the local economy, encourage sustainable growth, and improve the standard of living for locals (Davies and Evans 2019).

7 Cutting‐edge preservation technologies

In order to decrease food waste, increase the shelf life of perishable goods, and guarantee great quality for extended periods of time, innovations in food preservation are critical. Novel preservation approaches, from creative storage strategies to complex packaging solutions, have been developed as a result of technological advancements (Thapa Karki et al. 2021).

*Intelligent packaging solutions*: These solutions make use of materials that can react to environmental or food‐related changes. For instance, perishable goods' shelf life can be greatly increased by packaging that has the capacity to absorb moisture or excess oxygen. Similarly, when a product's expiration date approaches, indications that change color assist consumers in making smarter food choices, which lowers waste (Khan et al. 2021).

- Modified atmosphere packaging (MAP).

MAP modifies the air composition within a food package. Through the reduction of oxygen and the increase of other gases, such as carbon dioxide or nitrogen, MAP can slow down the spoiling process. Especially for fresh meats, dairy products, and produce, this method works well (Kalpana et al. 2019).

ii Atmospheric control.

Controlled atmosphere storage, which greatly lowers post‐harvest losses, includes adjusting the air composition in storage facilities. It is used for the bulk storage of fruits and vegetables (Murugesan et al. 2020).

iii Technologies for cooling and freezing.

Blast freezing and rapid cooling help preserve freshness, increasing food's shelf life without compromising quality. Techniques like vacuum cooling and cryogenic freezing are more effective and consume less energy than conventional techniques, which benefit the environment and the economy (Chauhan et al. 2019).

iv Ultrasonic and high‐pressure processing (HPP).

New technologies such as HPP and ultrasonic processing provide non‐thermal substitutes for traditional food preservation techniques. While maintaining the nutrients, flavor, and texture of the food, these methods can eliminate infections and spoilage organisms. Juices, sauces, and other liquid items benefit greatly from these techniques, which prolong their shelf life without requiring refrigeration or preservatives (Bland et al. 2018).

These cutting‐edge preservation technologies have limitations in terms of cost, scalability, and market adoption, notwithstanding their potential. Businesses may need to make a sizable initial investment, and some technologies require governmental approval. Nonetheless, more help and incentives are being provided as governments throughout the world start to realize how critical it is to combat food waste. These incentives include financing for research into new technology and laws that make food donation easier, as well as financial assistance for companies putting food waste reduction policies into practice (Rawson 2016).

## 22 Reducing Food Waste Through Digital Platforms

1. Using mobile applications to address food surplus

Mobile applications created to prevent food waste have made it possible for technology in the digital age to link excess food with those who can utilize it. These apps are minimizing food waste at the retail and consumer levels by bringing together food suppliers, restaurants, supermarkets, and customers directly (Balapour et al. 2020).

Apps such as “Too Good to Go,” for example, let users buy extra food from bakeries, restaurants, and supermarkets at a discount. In a similar vein, “Olio” links nearby companies and neighbors so that excess food can be shared rather than thrown away. These platforms give customers the chance to save money, find new local restaurants and items, and keep edible food out of landfills. To increase their impact, some applications also let users contribute food straight to food banks or non‐profit organizations (Mourad 2016).

These mobile applications have a big and expanding impact. As an illustration of the worldwide scalability of such solutions, “Too Good To Go” estimates preventing millions of meals from going to waste in multiple nations. Millions of food items have been shared among community members thanks to “Olio,” which has reduced food waste and promoted a sense of community. These achievements demonstrate how technology can significantly impact the reduction of food waste on a broad scale (de Souza et al. 2021).

Despite their success, these apps face challenges, including low user engagement, expanding the number of participating businesses, and ensuring the quality and safety of shared food. There is also the challenge of making the technology accessible to those in less developed regions where food waste is a critical issue, but smartphone penetration is lower. However, by continuing to innovate and adapt, mobile applications can play a crucial role in the global effort to reduce food waste (Ozdemir et al. 2025).

2 Emerging technologies and food waste

In the future, cutting‐edge technologies like blockchain, the Internet of Things, and artificial intelligence (AI) will be crucial to reducing food waste. Predictive analytics for inventory control and demand forecasting can be improved by AI and machine learning, increasing the effectiveness of food delivery networks. By monitoring food storage conditions in real time, IoT devices can prevent food from spoiling while being transported and stored. Blockchain technology ensures accountability and lowers losses by providing a transparent and safe method of tracking food goods across the supply chain (Visschers et al. 2016).

3 Demand forecasting with predictive analytics

Retailers and restaurants can determine product demand with greater accuracy by using predictive analytics to examine past sales data, weather trends, and consumer behavior trends. This minimizes waste by lowering the possibility of placing excessive orders or manufacturing food that is unsold.

4 Real‐time inventory management

These systems can notify companies when goods are about to expire, enabling thoughtful donations or promotions to cut down on waste. Food items may be tracked across the supply chain using RFID (radio‐frequency identification) technology and Internet of Things sensors, which can yield useful information for maximizing stock levels (Pagani and Pardo 2017).

5 Supply chain optimization

By examining transportation routes, storage conditions, and consumption habits, businesses can find inefficiencies and make adjustments that minimize food loss and spoilage. Rerouting deliveries to prevent delays, for instance, might guarantee that perishable goods arrive at their destination in the best possible condition (Damsbo‐Svendsen et al. 2017).

Technology is only one aspect of the fight against food waste; another is altering our attitudes toward and behaviors around food. It involves appreciating the resources used to produce our food and understanding how our daily decisions affect society and the environment. The road is difficult and calls for cooperation, creativity, and perseverance, but the benefits—a healthier world and a sustainable food system—make the effort worthwhile (Fraccascia and Nastasi 2023).

## 23 Case Studies and Best Practices

Food waste is a critical global issue. Approximately 30%–40% of food produced is discarded annually, leading to significant environmental, economic, health and social consequences. Various regions have implemented innovative strategies to mitigate food waste. Below are detailed case studies and best practices from around the world, highlighting successful interventions in Europe, America, Asia, and Africa.

### 23.1 Europe

Across Europe, various strategies have been effective in combating food waste. Denmark's *Too Good To Go* (TGTG) mobile application allows customers to purchase surplus food from restaurants and retailers at a discount, saving millions of meals from the bin and generating awareness of food waste; this aims to reduce carbon dioxide (CO_2_) emissions by saving meals (Lewandowski 2023). Also, in Hungary, Project Wasteless has achieved a 27% reduction in avoidable household food waste through national campaigns and educational outreach (Szabó‐Bódi et al. 2018; Kasza et al. 2020). Furthermore, in Belgium, Bruges implemented a *Zero Food Waste* strategy in healthcare facilities, with staff training and stock management improvements, reducing waste by 43% (Hardy and Desmet 2021). Meanwhile, Germany's *Unverpackt* stores promote package‐free shopping by encouraging customers to bring reusable containers and cutting food and packaging waste at the source (Zero Waste Europe 2023). These initiatives highlight Europe's leadership in combining technology, education, and behavior change to reduce food waste.

Europe's experience highlights the strength of combining policy support, public awareness, and innovation. Apps like *Too Good To Go* and programs like *Project Wasteless* show that easy‐to‐use technology and education can drive change at both household and business levels. As seen in Belgium's healthcare strategy and Germany's zero‐waste retail, government involvement shows how regulations and civic engagement work hand in hand. Therefore, these strategies can be scaled in other regions with strong digital systems and government support. Mobile apps and national campaigns can thrive in middle‐ and high‐income countries with existing waste management systems. However, success may depend on tailoring programs to cultural norms and logistical realities in different locations.

### 23.2 America

Large‐scale and grassroots initiatives are driving food waste reduction across North America. In the U.S., *The Farmlink Project* has redirected more than 130 million pounds of surplus farm produce to food banks since 2020, closing the gap between excess and hunger (Friedman and Ranganathan 2024). In Massachusetts, *Divert Inc*. has used anaerobic digestion to turn unsold food into renewable energy, repurposing over 2.3 billion pounds of waste since 2007 (Burch 2024). *412 Food Rescue*, based in Pittsburgh, connects volunteers with surplus food rescue opportunities via a mobile application, saving over 3 million pounds from landfills (Pilot 2024). On the West Coast, the *Pacific Coast Food Waste Commitment*, a public‐private collaboration, partnered with retailers to cut unsold food by 30% in just a few years (Litter 2018). Thus, these efforts showcase the scalability of both community‐driven and cross‐sector strategies.

U.S. case studies emphasize collaboration, tech‐driven logistics, and community involvement. Initiatives like *412 Food Rescue* and *The Farmlink Project* effectively use digital platforms to connect surplus food to people in need. Meanwhile, efforts like the *Pacific Coast Food Waste Commitment* show that large‐scale progress is possible when the private sector aligns with policy. In conclusion, these models can be adopted in countries with strong civil society and digital infrastructure. Urban areas, where surplus and need often coexist, are ideal. Expansion requires cold chain systems, data infrastructure, and partnerships with NGOs or government bodies.

### 23.3 Asia

Asia is applying both modern technology and traditional practices to address food waste. In Indonesia, *Gita Pertiwi* empowers communities with composting and food redistribution solutions to sustainably manage household and market waste (AP, G 2024). The *Sustainable Food Initiative* by Futouris, piloted in Asian hotels, introduced portion control and staff education, reducing weekly waste by up to 400 kg (Lee and Huang 2023). A cross‐country study of Asian restaurants also confirmed that awareness and staff training are critical to minimizing kitchen waste (Chia et al. 2024; Reitemeier et al. 2021). These projects reflect a wide range of solutions contributing to food system sustainability in the region.

Asia shows the importance of staff training, community‐led efforts, and practical, localized solutions. Projects in Indonesia demonstrate that composting and hotel waste audits can significantly reduce waste. Adding food waste education into tourism and hospitality also helps raise awareness. Thus, these community‐led and hospitality‐based models are adaptable in urban and semi‐urban areas. They are affordable and can be integrated into public health and environmental programs. Successful expansion depends on local buy‐in, training, and modest investment in tools or technology.

### 23.4 Africa

Digital innovation has been on the increase in recent years, and it has opened up new ways to cut down on food waste in Africa. For example, technology has helped close gaps in supply chains, made it possible to redistribute food in real time, and made it easier for people to go to markets (FAO 2023). Increasingly, digital platforms are being utilized to connect food producers, merchants, and consumers. This helps save extra food from going to waste all across the continent.

Using mobile platforms and applications to quickly match surplus food with customer demand is one of the most effective ways to do this. The Twiga Foods platform in Kenya, for example, employs data‐driven logistics to connect farmers directly with merchants. This cuts down on the losses that happen after harvest because of bad market connections and slow shipping (Twiga Foods 2023). Twiga has been able to decrease post‐harvest losses of fruits and vegetables by as much as 30% (Chege et al. 2023) by using predictive algorithms and real‐time surveillance. Moreover, in Nigeria, the Chowberry app does the same thing by moving food that has not sold but is still safe to eat from stores to charities and low‐income communities at lower costs. The technology works with inventory management systems to mark things that are about to expire, which helps keep food secure for people who are susceptible (Schneider and Eriksson 2020). These digital advancements also help with data gathering and analysis, which are necessary for keeping an eye on food waste trends and improving food recovery. Stakeholders may use digital dashboards to keep an eye on the flow of food through different parts of the value chain, pinpointing key sites of loss and influencing targeted actions (FAO 2023).

Digital food waste platforms in Africa have a lot of potential, but they have certain problems with scaling. For example, internet connectivity is restricted in remote regions, smartphones are expensive, and the cold chain infrastructure is insufficient. However, the tremendous rise in mobile phone use and the growing investments in agricultural technology throughout the continent show that there is a lot of room for development. International development organizations and businesses are starting to work with local entrepreneurs to improve their technological skills and support digital ecosystems that include everyone and are focused on reducing food waste (FAO 2023). In general, using digital platforms in Africa offers a game‐changing chance to cut down on food waste, make distribution more efficient, and make the food chain more resilient. Digital solutions may help solve food insecurity and promote sustainable food systems across the continent, but only if they are backed up by laws and investments in infrastructure.

## 24 Challenges and Barriers

1. Poor infrastructure (cold storage, transportation, and garbage collection)

Food is lost in many low‐ and middle‐income countries, especially in sub‐Saharan Africa and South Asia, because of a lack of post‐harvest infrastructure (Stathers et al. 2020). Perishables often spoil because there is insufficient refrigeration, roads are in poor condition, and access to processing facilities is limited. In Tanzania, for instance, nearly 48% of food is lost after harvest because of poor storage and transport (UNDP 2023).

2 Consumer behavior and cultural attitudes

At the household level, food waste happens because of overbuying, confusion over “best‐before” dates, or personal preferences for how food looks. In high‐income countries, cultural expectations for perfect‐looking produce contribute to higher waste. In some cultures, serving too much food is seen as a form of hospitality, further encouraging waste (FAO 2023).

3 Limited policy and regulatory environments

Many nations lack a national strategy, legal mandates, or regulatory pressure to reduce food waste. Without clear policies, action remains voluntary. France has legislation banning supermarket food waste, but most African countries do not have similar laws (Zero Waste Europe 2023).

4 Perceived costs and economic disincentives

Businesses may see food donation or reduction efforts as costly or impractical. Logistics, storage, and technology costs often make these actions feel financial, especially for small and medium enterprises (ReFED 2023).

5 Lack of education and awareness

There is limited public knowledge about food waste's environmental and social impacts. Many people, including consumers and supply chain actors, do not realize how their choices contribute to the problem. Studies in parts of Africa show that traders and vendors often do not see food waste as urgent (UNEP 2023).

6 Deficient data and monitoring systems

Most countries lack reliable data on food waste at the household, retail, and informal sector levels. Without data, it is hard to plan, track, or measure interventions effectively—especially in rural areas and informal markets where most food transactions occur (FAO 2023).

7 Fragmented supply chains and inadequate coordination

In many developing economies, supply chains are long, informal, and disjointed. Producers, processors, and retailers often do not share goals or practices. This poor coordination creates supply–demand mismatches that cause waste before food reaches consumers (WRI 2023b).

8 Technological limitations

Advanced tools like innovative packaging, AI inventory tracking, or food‐sharing platforms can help reduce waste—but they are expensive and often unavailable in most countries. Small farmers and vendors in Asia and Africa lack the digital infrastructure to use these innovations (GAIA 2023).

## 25 Apps Preventing Food Waste

A variety of applications available through digital information systems may have an impact on sustainability. By decreasing food waste and enhancing the long‐term sustainability of the food sector, this transition to digital technology has sped up the development of digital solutions. Generally speaking, by creating social and environmental value, digital technologies can increase the sustainability of a digital business. In Turkey, for instance, a number of food waste mobile applications, including Yenir, Oreka, Raf, Sifir, and others, were developed to assist food businesses in selling food that would otherwise be thrown away (Elkhalifa et al. 2019).

Businesses can both make money and give surplus food to willing customers by using mobile apps that prevent food waste. Mobile apps demonstrate how technology may help the food industry benefit consumers, companies, the environment, and society. To cut down on food waste, there are numerous apps that implement various strategies. Some concentrate on reducing household waste, whereas others specialize in offering an opportunity to creatively repurpose excess food (Vo‐Thanh et al. 2021).

There are three types of apps that help avoid food waste

- Apps to reduce household food waste
- Apps for purchasing discounted food
- Apps for giving away extra food to people in need

### 25.1 Apps to Reduce Household Food Waste

The apps listed below are for individual use and help reduce food waste at home. They keep track of expiration dates and ensure that the food is consumed before it goes bad (Kuźniar et al. 2025).

- No Waste

Food expiration dates in your cupboard and refrigerator are tracked by the No Waste app. In addition to manually entering the expiration dates of your food, the app lets you enable notifications to be notified when your food is going to expire. To assist you (and everyone else on the app) in staying informed, No Waste offers a social media community where you can post your food waste statistics. The location is anywhere.

b OLIO

Neighborhoods may work together to combat food waste and expiration by using the OLIO app. In order for you and other community members to post food that they no longer want, encourage your neighbors to download the platform. Additionally, this app is a great method to interact with your neighborhood. The location is anywhere.

c Kitche

Kitche is a fantastic program that keeps you from inadvertently buying something you previously owned at home. You can use Kitche to keep track of the food you have at home by taking a picture of your grocery receipt. Additionally, the app is tailored to you, suggesting dishes according to your dietary restrictions. The location is anywhere.

### 25.2 Apps for Purchasing Discounted Food

Businesses can use the following applications to find customers who can either eat or dispose of their almost‐expiring food.

- Flash food

The Flash food app locates groceries near you nearing their expiration dates that can be purchased at a significantly discounted rate. You can pay through the Flash food app too. This app is excellent for anyone who is looking to snag good food for cheap. The location is US states and Canada, but it is expanding.

b Food for All

Restaurants may interact with consumers and serve food that would otherwise be thrown away by using the Food for All app. You can buy the meals an hour before the restaurants close, and the savings are frequently as high as 80%. To view participating stores in your area, download the app and enter your location. You can also give food to people in need through the app. There are more than 200 restaurants in Boston and New York.

c Too Good To Go

In an effort to reduce food waste, Too Good To Go lists local restaurants, bakeries, cafes, and shops that sell their unsold food at a reduced price. More than 70 million meals have been rescued globally by Too Good To Go, a Certified B Corporation. Using the app, you may place a direct meal order, pick it up, and pay a small portion of the cost. New York and Denmark are the locations, but they are growing.

d Your Local

Another app that focuses on food conservation is Your Local. By enabling consumers to pick up excess food from restaurants and supermarkets for a fraction of the price, it helps communities avoid food waste. Only Denmark and New York have access to the relatively new software.

e Karma

Food surpluses are being sold by Karma at steep discounts. Get the app for food waste and check out your options! Additionally, takeaway is available from over 20,000 restaurants throughout Europe, the UK, Denmark, France, and Sweden.

f Phenix

Phenix is for both consumers (buying food at a discount) and companies (selling food that didn't sell). Locate the companies in your neighborhood that sell anti‐waste baskets composed of their daily unsold inventory. It might be anything from grocery stores and supermarkets to bakeries, restaurants, caterers, and more. On average, there is a 50% price reduction.

More than 15,000 retailers in the following cities have Phenix as a partner: Reims, Caen, Quimper, Tours, La Rochelle, Limoges, Perpignan, Chambéry, Strasbourg, Dijon, Besançon, Montpellier, Toulouse, Bordeaux, Marseille, Lille, Paris, Lyon, Rennes, Nantes, Grenoble, and Toulouse. If you're from Belgium, Spain, Portugal, Italy, France, or Europe, you can also utilize it (Teigiserova et al. 2020).

g Go Mkt

You've undoubtedly heard that supporting your town by shopping locally is a terrific idea. Go Mkt advises you to patronize nearby eateries and stores that have extra inventory. To let you know about local product options, the app can even send push notifications to your mobile. New York is the setting, but it is growing.

h Imperfect Foods

Food that is visually unpleasant is also worthy of love. Whether it is because of their appearance or because they have extra, Imperfect Foods is a subscription box business that delivers reasonably priced groceries to you.

Despite the fact that the food is excellent and totally edible, supermarket shops' stringent policies often only permit certain foods to be displayed on their shelves. What is the best? You can remove the items you do not want and see what will be delivered to you before it is sent. It is spread throughout 41 US states, including the majority of the West South‐Central area (Visschers et al. 2016).

i Misfits Market

Misfits Market was founded to provide a place for unusual‐looking products, much like Imperfect Foods. In order to identify inefficiencies in the food system and sell food that would otherwise go to waste, the company works closely with farmers and food producers. They deliver their products straight to your door and sell them for up to 40% less than grocery store costs. There are 44 US states.

j Hungry Harvest

Delivery: The Hungry Harvest saves and sells reasonably priced farm‐fresh fruits and vegetables that would otherwise go to waste throughout the United States (Maryland, Washington, DC, Virginia, Greater Philadelphia, Southern New Jersey, Northern Delaware, South Florida, The Triangle Area, and Charlotte, North Carolina).

At least 10 pounds of food are kept out of the landfill with each delivery. Additionally, Hungry Harvest provides food to underprivileged local organizations. More than 27 million pounds of produce have been saved by Hungry Harvest since 2014, and more than 1.7 million pounds have been given to those in need. Metro Area of Detroit.

### 25.3 Apps for Giving Away Extra Food to People in Need

One of the most disturbing aspects of the food waste crisis is that millions of people going hungry could use that food. These apps help do justice by getting leftover food to those who need it.

- Food Rescue US

The Food Rescue US app links companies that have extra food to organizations that fight hunger. Additionally, it makes it easier for volunteers to move the food from one place to another. Whether you want to volunteer to help carry food or register your company, this app is a terrific way to reduce food waste and give back to the community. As of right now, the Food Rescue US app has prevented over 75 million meals from ending up in landfills and reaching the people who need them the most. The 21 US states are the place.

b Food Rescue Hero

Through the transportation of extra food from point A to point B, this app assists volunteers in serving their community. It directs people to the nearby non‐profits that serve food and connects them with them. Anytime the user has free time, they can perform a “rescue.” To help “rescuers” learn more about the organizations they are helping, the app also offers background information on each non‐profit. It is in the United States.

c Raeri (formerly Transfernation)

Through this app, users may connect with philanthropic organizations that provide leftover food from important events. They redistribute food to those in need after gathering it. In order to guarantee that the food is picked up and delivered, the app links volunteers, drivers for Uber, and drivers for Lyft with the event organizers. New York serves as its base.

d No Food Waste

This app, developed in India, uses crowdsourcing to identify “hunger spots” throughout the country and redistribute excess food to such locations. The No Food Waste app team will pick up and deliver the food to hunger places if it can feed more than 50 people. If the food is enough to feed fewer than 50 people, users drop it off at specific hunger places (identified on the app). It is based in India (Asia).

e Yo No Desperdicio

A food waste app called Yo No Desperdicio is based in Spain. People share food they no longer need, along with information about it. Users can arrange a meeting to pick up the products and possibly even trade them if they see anything they like. In essence, the app is a food waste prevention social media app.

## 26 Emerging Trends in Food Waste Reduction and Opportunities for Interdisciplinary Research

Emerging trends in food waste reduction are increasingly leveraging advanced technologies, notably artificial intelligence (AI), to enhance efficiency and effectiveness. AI‐driven systems are being deployed to optimize supply chain management, predict demand more accurately, and identify surplus or substandard products before they spoil, thereby minimizing waste (Elgalb and Gerges 2024). For instance, machine learning algorithms can analyze historical data to forecast consumption patterns, enabling retailers and producers to adjust inventory levels proactively. Additionally, AI‐powered image recognition tools are used to assess food quality and expiration dates, reducing the likelihood of discarding edible food. These innovations not only streamline operational processes but also contribute to sustainability goals by significantly decreasing the volume of food wasted. However, despite their promise, the integration of AI into food waste reduction faces challenges related to data privacy, implementation costs, and technological accessibility, particularly in low‐resource settings. As such, although AI presents transformative opportunities, its deployment must be critically evaluated to ensure equitable and practical solutions.

The opportunities for interdisciplinary research in food waste reduction are both extensive and vital for addressing this complex issue in a holistic manner. Effectively tackling food waste demands insights from various fields, including environmental science, economics, sociology, technology, and policy studies. For instance, environmental scientists can evaluate the ecological impacts of different waste reduction strategies, whereas economists examine market incentives that drive waste‐related behaviors. Sociologists and behavioral psychologists can investigate consumer attitudes and cultural practices that contribute to food wastage, thus informing more impactful public education campaigns. Additionally, collaboration with technologists can yield innovative solutions, such as AI applications or sensor technologies, that enable more efficient monitoring and management of waste. Policy researchers can contribute by developing frameworks that promote waste reduction at multiple levels. Interdisciplinary research cultivates a comprehensive understanding of the diverse causes and consequences of food waste, allowing for the creation of integrated solutions that are socially acceptable, economically feasible, and environmentally sustainable. Ultimately, such cross‐disciplinary collaboration is essential for devising scalable, context‐specific strategies that can effectively reduce food waste on a global scale.

## 27 Problems and Challenges

A significant issue is the inadequate infrastructure, particularly in low‐ and middle‐income nations, where insufficient cold storage, ineffective transportation systems, and restricted market facilities result in substantial post‐harvest losses. For instance, in Tanzania, nearly 48% of food is wasted post‐harvest because of inadequate storage and transportation systems. This illustrates the significance of investing in supply chain systems. In addition to infrastructure, consumer behavior and cultural attitudes remain significant factors in food waste. Individuals discard more food at home and in retail establishments because of misinterpretation of food labels, a preference for aesthetically flawless produce, and adherence to cultural norms regarding the excessive presentation of food.

Moreover, deficiencies in policy and governance exacerbate challenges. Numerous countries lack explicit national strategies, enforceable legislation, or robust mechanisms for ensuring accountability in the systematic management of food waste. Despite the presence of stringent legislation, enforcement is frequently inconsistent, and convoluted donation regulations often hinder the distribution of surplus safe food. Furthermore, stakeholder practices such as inadequate handling, substandard packaging, and insufficient worker training contribute to spoilage throughout the supply chain.

In addition, technological and logistical issues persist. Digital innovations, such as Too Good To Go, Olio, and Twiga Foods, exhibit potential; however, their implementation is hindered in regions characterized by inadequate connectivity, minimal smartphone penetration, and deficient cold chain infrastructure. The absence of data and measurements impedes the formulation of evidence‐based policies. The FLW Accounting and Reporting Standard provides a global framework; however, its adoption is limited because of poor data quality, insufficient reporting, and inadequate institutional capacity in developing regions.

## 28 Research and Policy Gaps

This review identifies essential gaps that need to be addressed to make a meaningful difference in food waste. First, there is limited data specific to the context in Africa, Asia, and Latin America. This means that interventions are often introduced from high‐income areas, where they do not align well with the local culture or the way things operate. Second, there is a lack of research on the psychological and behavioral factors that lead to food waste in different cultures. Third, there is a lack of longitudinal studies examining the effectiveness of awareness campaigns, technologies, or policies in assessing the impact of interventions. Furthermore, the incorporation of circular economy strategies, such as the secure recycling of waste into animal feed, compost, or energy, remains inadequate in both policy and implementation. There is not enough research on how throwing away food affects health, especially in low‐income areas where waste breaks down and causes diseases that spread through water and bad sanitation. Lastly, there is a lack of research on and limited use of economic tools and private sector incentives to reduce waste, such as tax breaks or fines.

## 29 Recommendations for Future Research and Policy

- Enhance Data Systems

Develop national and regional food waste accounting systems tailored to local conditions, supported by capacity‐building initiatives and digital monitoring technologies (including AI, IoT, and blockchain).

ii Put Developing Countries First

Do in‐depth research in Africa, Asia, and Latin America, focusing on differences between urban and rural areas, cultural factors, and how the local market works.

iii Utilize interdisciplinary methods

Combine food science, sociology, public health, and environmental studies to gain a comprehensive understanding of the complex factors that drive and influence food waste.

iv Evaluate and scale interventions

Do long‐term studies on how well campaigns, digital platforms, and policy tools work over time, and then use successful models in more places.

v Advance Circular Economy Models

Make it easier, safer, and cheaper to turn food waste into feed, fertilizer, bioenergy, and bioplastics, with clear rules to support them.

vi Strengthen Governance and Enforcement

Incorporate food waste into national strategies for food security, climate change, and health, and streamline enforcement to ensure compliance.

## 30 Novelty and Research Gap

A multitude of reviews and reports have investigated FLW; however, many remain constrained in scope, focusing solely on technical interventions such as improvements to the cold chain, consumer behavior in affluent countries, or specific phases of the supply chain, including post‐harvest or retail stages. Although these initiatives are beneficial, they fail to address significant deficiencies in the literature. The majority of existing studies primarily concentrate on Europe, North America, and certain regions of Asia. Evidence from Sub‐Saharan Africa and other developing regions, where infrastructure issues and cultural disparities are significantly more evident, is limited. Secondly, there is considerable discourse surrounding technological solutions; however, insufficient attention is given to the matters related to policy, governance, and enforcement, particularly in regions where inadequate regulatory frameworks exacerbate food waste. The environmental, social, economic, and health dimensions of FLW are often examined in isolation, rather than within a holistic systems framework that integrates sustainability, food security, and public health outcomes. Moreover, despite the existence of the FLW Standard, its applicability and challenges in low‐resource settings remain inadequately explored. Similarly, the potential of circular economy models, such as converting food waste into feed, compost, or energy, and emerging digital innovations, including artificial intelligence, the Internet of Things, and mobile redistribution platforms, have not been adequately incorporated in the existing literature. This review's originality lies in its thorough, multifaceted analysis that incorporates evidence related to environmental, economic, social, and health impacts; highlights case studies from both developed and developing regions, with a particular emphasis on Tanzania and Sub‐Saharan Africa; and critically assesses policy and governance challenges alongside technical interventions. This review characterizes food waste as a technical challenge as well as a socio‐political and public health concern, thereby fostering a thorough and globally inclusive understanding of food waste reduction while outlining clear avenues for future research and initiatives to achieve SDG 12.3.

## 31 Conclusion

FLW is one of the most harmful yet preventable problems in today's food systems. There are several benefits to reducing FLW that are not just good for the environment, but also good for the economy, society, and health. When food is thrown away, the land, water, labor, and energy that went into making it are also lost. This makes the ecosystem worse and adds a lot to climate change. Food waste costs the economy billions of dollars every year. It also makes food insecurity and nutrition gaps worse, especially in areas with low incomes and food shortages. When food waste is thrown out in the wrong way, it can spread illness and pollute water supplies, which is bad for public health.

Moreover, the review demonstrates that there are long‐term solutions, and many nations and areas have put in place successful ways to cut down on food waste that may be used as models. However, there are still big problems, such as regulations that are not clear, not enough people knowing about them, not enough technology, and problems with getting food to people and getting it back. Governments, businesses, research institutes, and civil society will all need to work together to get rid of these problems. To get the most out of FLW reduction, we need to put money into infrastructure, data systems, education, and making sure that policies are consistent. In the end, fighting food waste is not only the right thing to do, but it is also a key part of attaining sustainable development, climate resilience, and global food security.

## Author Contributions

**Warren Kilemile:** conceptualization (equal), methodology (equal), resources (equal), writing – original draft (equal), writing – review and editing (equal). **Fabian Mihafu:** conceptualization (equal), methodology (equal), supervision (equal), writing – original draft (equal), writing – review and editing (equal). **Kelvin E. Vulla:** conceptualization (equal), resources (equal), visualization (equal), writing – original draft (equal), writing – review and editing (equal). **Vidhya Chandrasekaran:** conceptualization (equal), methodology (equal), supervision (equal), writing – original draft (equal), writing – review and editing (equal).

## Conflicts of Interest

The authors declare no conflicts of interest.

## Data Availability

The data that support the findings of this study are available from the corresponding author upon reasonable request.
